# Flotation Separation of Diaspore and Kaolinite by Using a Mixed Collector of Sodium Oleate-Tert Dodecyl Mercaptan

**DOI:** 10.3389/fchem.2019.00813

**Published:** 2019-12-12

**Authors:** Xiaofei Man, Leming Ou, Chenliang Wang, Saizhen Jin, Xiqi Ma

**Affiliations:** School of Minerals Processing and Bioengineering, Central South University, Changsha, China

**Keywords:** flotation, diaspore, kaolinite, mixed collector, sodium oleate, tert dodecyl mercaptan

## Abstract

Sodium oleate (NaOl), a collector in diaspore flotation, has been widely used for more than 30 years, while its low selectivity becomes an issue under today's process requirement. This study introduced tert dodecyl mercaptan (TDM) together with NaOl as a mixed collector to improve selectivity in diaspore flotation. We found that using the mixed collector of NaOl/TDM (total concentration 0.1 mM, the molar ratio 8:2 of NaOl: TDM) at pH = 9–10 significantly effectively separated diaspore and kaolinite. Comparing the recovery of Al_2_O_3_ and the ratio of Al_2_O_3_ to SiO_2_ (A/S) treated by NaOl/TDM (pH = 9) and NaOl (pH = 10), the Al_2_O_3_ recovery and A/S in concentrate for NaOl/TDM are 7.5% and 2.2 higher than that for NaOl in mixed mineral flotation. Also, surface tension measurements, Zeta potential measurements and Fourier Transform Infrared (FTIR) spectra analysis were used to examine its selectivity from a flotation mechanical perspective. Surface tension measurements show that mixed collector NaOl/TDM has stronger surface activity and hydrophobic association than NaOl. The results of Zeta potential measurements and FTIR spectra analysis indicate that NaOl and TDM can selectively co-adsorb diaspore through physical adsorption. Moreover, the adsorption of TDM promotes the adsorption of NaOl on diaspore. However, when NaOl/TDM treats on kaolinite together, TDM can hardly adsorb on mineral surface, nor can it promote the adsorption of NaOl.

## Introduction

Bauxite is an important raw material for industrial production of alumina (Zhang et al., 2017a, 2018a). In China, diaspore is the primary mineral in bauxite, accounting for more than 98% of the total reserves (Zhang et al., 2016). China's bauxite resources have the characteristics of high alumina, high silicon, and low mass ratio of Al_2_O_3_ to SiO_2_ (A/S) (Liu et al., 2007; Hong et al., 2008; Jiang et al., 2012). Bauxite with A/S ratio of 4–6 accounts for about 60% of the total (Liu et al., 2007; Jiang et al., 2010a). However, through Bayer process, the principal method of producing alumina from bauxite (Zhang et al., 2019), producing alumina is that the mass ratio of A/S should be more than 8 (Jiang et al., 2010b; Gibson et al., 2017). Therefore, it is necessary to pre-desilicate bauxite to improve its mass ratio of A/S.

Kaolinite is a common layered silicate mineral (Jing et al., 2015). As one of the main gangue-bearing minerals in bauxite, the removal of kaolinite is of great significance to the desilication process of bauxite (Jiang et al., 2014; Liang et al., 2017). At present, there are many desilication methods. Currently, flotation desilication is the most effective way to separate diaspore from kaolinite (Jing et al., 2015; Zhang et al., 2017b), especially direct flotation (floating diaspore but suppressing kaolinite desilication) has been widely used in practical production (Zhang et al., 2018b). Flotation reagents play an important role in the flotation process (Jiang et al., 2010b). In recent years, various surfactants have been used as collectors for selective separation of diaspore and kaolinite in direct flotation, such as oleic acid (Huang et al., 2012), tall oil, oxidized paraffin soap (Yuehua et al., 2004; Hu et al., 2005), 733, styrene phosphate, hydroxamic acids, propyl gallate (Lyu et al., 2019) and their mixtures (Jiang et al., 2014), and novel synthetic chelates (Deng et al., 2015, 2016). The selectivity of synthetic chelating collectors is stronger than that of oleic acid collectors (Gibson et al., 2017), and their functional groups are mainly composed of one or several carboxyl groups, hydroxamic acid salts and amides (Jiang et al., 2011). However, oleic acid collectors are still widely used in bauxite flotation as the most commonly used collectors (Deng et al., 2015; Wang et al., 2017) due to their low cost, high collection capacity and low toxicity in China (Jarek et al., 2010; Chernyshova et al., 2011). But the strong collecting ability and low selectivity of oleic acid collectors may lead to low A/S ratio or low recovery of diaspore concentrate.

Thus, to enhance the selectivity of oleic acid collectors, the use of synergists has been increasingly investigated in recent years. The common synergists are mainly non-ionic surfactants (Kongolo et al., 2003). These non-ionic surfactants can promote the dissolution and emulsification of oleic acid collectors, reduce the surface tension of the solution, and strenigthen the adsorption of collectors on mineral surfaces (Cao et al., 2014). By adding synergists, the efficiency of collectors is improved and the dosage of collectors is reduced. Tert dodecyl mercaptan (C_12_H_25_SH, TDM), a novel surfactant, which has been used as a collector in sulfide ore flotation (sphalerite, arsenopyrite, and molybdenite) (Chen et al., 2010; Jiao et al., 2016), has great potential to be used as a synergist to improve the efficiency of diaspore flotation, despite the limitation of high price and complex production process (Jiao et al., 2016).

In this paper, the flotation separation of diaspore and kaolinite by mixing tert dodecyl mercaptan (TDM) with sodium oleate (NaOl) as a mixed collector is proposed for the first time. The flotation performance of diaspore and kaolinite was examined by single mineral tests. In addition, we analyzed the surface activity of the mixed collector by measuring its surface tension, and investigated the absorption mechanism between the mixed collector and mineral surfaces by analyzing Zeta potential and FTIR spectra.

## Materials and Methods

### Materials

The single minerals used in the experiments were diaspore and kaolinite from Hebei Province and Suzhou Province of China respectively. They were both higher than 90% pure based on X-ray diffraction (shown in Supplementary Material) and chemical analysis. Through handpicking massive ores, artificial crushing and grounding in a porcelain mill, minerals of +37–74 μm were screened out for single mineral flotation tests and mixed mineral flotation tests. The screened minerals were grinded to −5 μm by an agate mortar for Zeta potential determinations and FTIR spectra analysis.

### Reagents

Chemically pure sodium oleate (NaOl), chemically pure tert dodecyl mercaptan (TDM) and NaOl/TDM mixtures with different molar ratios were used as collectors. NaOl was supplied by China Pharmaceutical Group Chemical Reagent Co. Ltd. And TDM was supplied by Shanghai Macklin Biochemical Co. Ltd. All the reagents were ready-to-use to avoid drug failure. The analytical purity of hydrochloric acid (HCl) and sodium hydroxide (NaOH) were used as pH regulators of the system. Deionized (DI) water was used for all experiments.

### Flotation Tests

**Figure 1:**
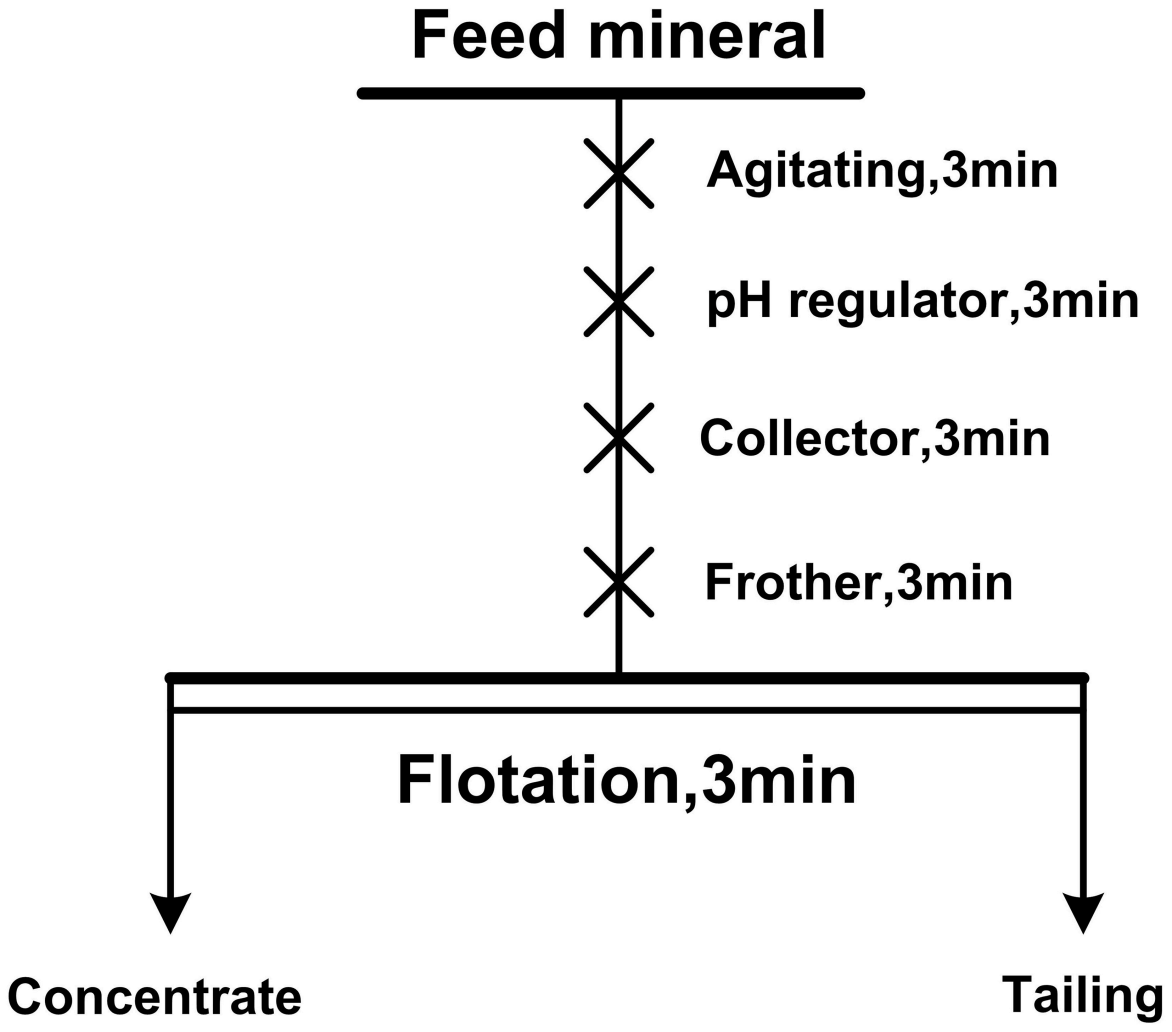
Flowsheet of the flotation tests.

Single mineral flotation tests and mixed binary mineral flotation tests were carried out in an XFG hitch groove flotation machine with a 40 ml plexiglass cell at a spindle speed of 1,900 rpm. The mineral suspension was prepared by 2.0 g of single or artificially mixed minerals to 35 ml of deionized water in a plexiglass cell. The mass ratio of diaspore to kaolinite in artificial mixed minerals is 3:1 and the A/S ratio of mixed minerals is 3.49. Figure 1 is the flow chart of the flotation test. For single mineral flotation, the flotation concentrates and tails were filtered, dried, and weighed, and the recovery was calculated. For mixed binary mineral flotation, the content of Al and Si in the flotation products was analyzed and A/S was calculated.

### Surface Tension Measurements

The surface tension was measured by the platinum ring detachment method with a BZY-2 automatic tensiometer. Before measurement, the platinum ring was cleaned with deionized water and alcohol and subsequently it was placed on the alcohol lamp flame to remove the remnants from the last measurement. When the surface tension of the collector was measured, equal volume solution was weighed and put into the glass dish of the surface tensiometer. The average of three tests was taken as the final test result.

### Zeta Potential Determinations

Zeta potentials of pure minerals before and after adding collectors were measured by Coulter Delsa-440SX Zeta potential analyzer (Brookhaven Corporation, USA) at 25°C. The suspension consists of 30 mg single mineral particles and 40 ml KCl (0.01 M) background electrolyte. After adjusting the pulp pH with HCl or NaOH, the collector was added. The suspension was agitated by a magnetic stirrer for 5 min and settled for 10 min. The supernatant was extracted by a syringe for Zeta potential measurement. The average of three tests was taken as the final result.

### FTIR Spectra Analysis

Infrared spectra of reagents and minerals before and after their interaction were determined by IRAffnity-1 Fourier Transform Infrared Spectrometer (shimadzu Japan). The single mineral particles (0.5 g) were added to 35 ml deionized water. After adjusting the pulp pH and adding reagents, the sample was stirred for 40 min and filtered for 40 min subsequently. After washing three times with distilled water at the same pH, the solid samples were dried in a vacuum oven at 35°C for 24 h and analyzed by FTIR spectra analysis.

## Results and Discussions

### Flotation Tests

**Figure 2:**
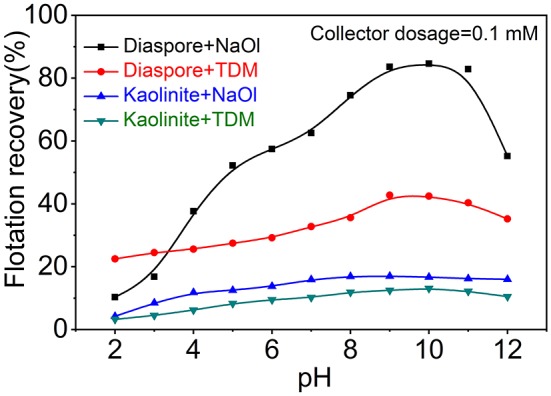
Flotation recovery of diaspore and kaolinite with NaOl or TDM as collector (Collector dosage = 0.1 mM) as a function of pH.

Figure 2 shows that flotation recovery of diaspore and kaolinite varies with pH when NaOl or TDM was used as a single collector. As shown in Figure 2, the recovery of diaspore using NaOl increases first and then decreases with the enhancement of pH. The best pH range for the flotation by NaOl is 9–11, which is consistent with the results of other scholars (Xu et al., 2014). The recovery of NaOl to diaspore reaches the maximum of 84.6%, but at this time the recovery of NaOl to kaolinite also reaches 16.7%, so single collector NaOl cannot make an efficient separation between diaspore and kaolinite. Unlike NaOl, recovery of diaspore and kaolinite with TDM as collector alone is relatively low. The maximum recovery of diaspore by TDM at pH = 9 is only 42.8%. Obviously, neither NaOl nor TDM alone can achieve efficient flotation separation of diaspore from kaolinite. We considered further investigating the flotation of diaspore and kaolinite with NaOl/TDM mixed collector in order to obtain better separation effect.

**Figure 3:**
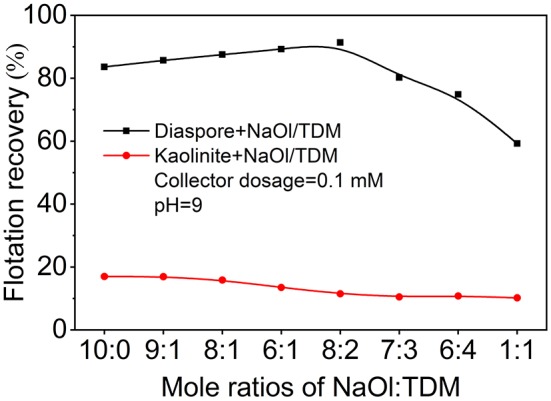
Effect of the molar ratio of NaOl to TDM (Collector dosage = 0.1 mM, pH = 9) on the flotation recovery of diaspore and kaolinite.

Considering the important influence of molar ratio on mixed collector, the effects of molar ratio of NaOl and TDM on the recovery of diaspore and kaolinite were investigated. Figure 3 shows that effects of different molar ratio of NaOl to TDM on the flotation recovery of diaspore and kaolinite. The effect of molar ratio of NaOl to TDM on the recovery of diaspore is obvious. When the molar fraction of TDM is less than 20%, the flotation recovery of diaspore with mixed collector is higher than that with NaOl. With the increase of molar fraction of TDM, the recovery of diaspore decreases sharply. When the molar ratio of NaOl to TDM is 8:2, the recovery of diaspore reaches the maximum of 91.4%. Unlike diaspore, the recovery of kaolinite does not change significantly with the molar ratio of mixed collector. The recovery of kaolinite decreases slowly with the increase of molar fraction of TDM. When the molar ratio of NaOl to TDM is 8:2, the recovery of kaolinite is 11.5%, which is 5.6% lower than that of single collector NaOl. Therefore, the optimum molar ratio of diaspore and kaolinite separated by mixed collector is about 8:2 for NaOl: TDM.

**Figure 4:**
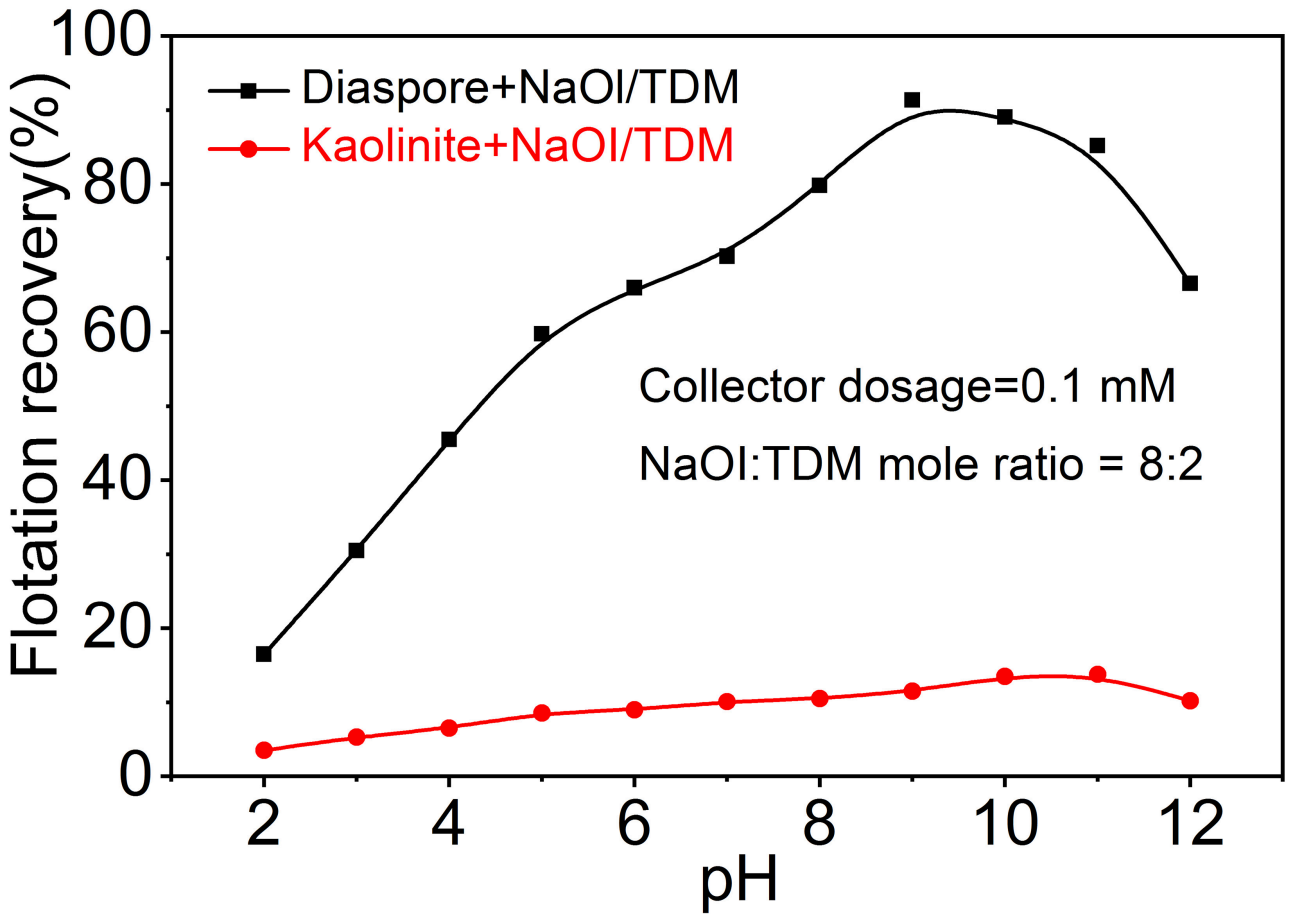
Flotation recovery of diaspore and kaolinite with the NaOl/TDM (dosage = 0.1 mM, NaOl: TDM mole ratio = 8:2) as a function of pH.

Figure 4 shows the effect of pH on the recovery of diaspore and kaolinite by flotation with NaOl/TDM mixed collector. The recovery of diaspore increases firstly and then decreases with the increase of pH. When the pH is 9, the recovery of diaspore reaches the maximum of 91.35%. Unlike diaspore, the recovery of kaolinite gradually increases from a minimum of 3.5% at pH 2 to a maximum of 13.8% at pH 11 and decreases with the increase until pH equals 12. Therefore, the optimal pH for the flotation separation of diaspore from kaolinite with mixed collector is 9.

**Figure 5:**
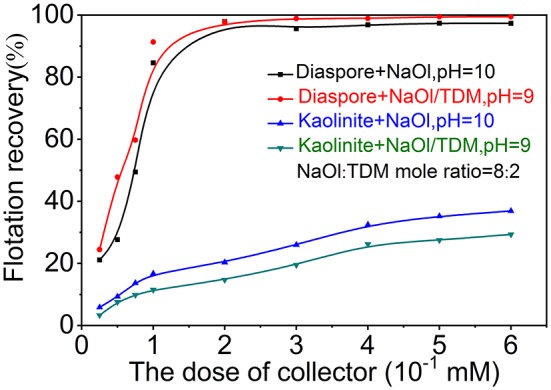
Effect of collector dosage on the diaspore and kaolinite flotation recovery with NaOl (pH = 10) or NaOl/TDM (pH = 9, NaOl: TDM mole ratio = 8:2) as collector.

The dosage influence of new collector NaOl/TDM on the flotation of diaspore and kaolinite was investigated by reference to NaOl dosage. Figure 5 presents the effect of collector dosage on the diaspore and kaolinite flotation recovery with NaOl or NaOl/TDM as collector. As shown in Figure 5, the flotation recovery of diaspore increases rapidly with the increase of collector dosage when the concentration of collector is less than 0.1 mM, and then increases slowly. The recovery of diaspore by mixed collector is higher than that by NaOl single collector at various concentrations. When the concentration of the collector is 0.1 mM, the recovery of NaOl and mixed collector for diaspore are 84.62 and 91.35%, respectively. For kaolinite flotation, the recovery of kaolinite increases with the concentration of collector. When the concentrate of collector is more than 0.075 mM, the recovery of mixed collector for kaolinite is much lower than that of NaOl. The recovery of kaolinite is 16.7% when the concentrate of NaOl is 0.1 mM. At this time, the recovery of kaolinite with mixed collector is only 11.5%. Therefore, compared with the single collector of NaOl, NaOl/TDM mixed collector has better separation effect on diaspore and kaolinite. The optimum dosage range of mixed collector is from 0.1 to 0.15 mM.

**Figure 6:**
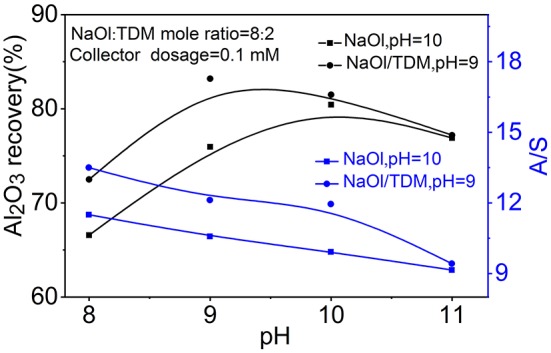
The separation results of mixed binary minerals as a function of pH with NaOl (dosage = 0.1 mM, pH = 10) or NaOl/TDM (dosage = 0.1 mM, NaOl: TDM mole ratio = 8:2, pH = 9) as collector.

In order to further verity the flotation effect of NaOl/TDM mixed collector, the flotation effect of mixed binary minerals at the special pH was studied. Figure 6 shows the separation results of the mixed minerals as a function of pH. The A/S of the concentrate with NaOl/TDM decreases from 13.5 to 9.3 as the increase of pH. Meanwhile, the Al_2_O_3_ recovery increases from 73.2 to 83.6% by increasing pH from 8 to 9, and then decreases to 76.5% at pH 11. When NaOl is used as collector, the A/S decreases from 11.5 to 9.1 and the Al_2_O_3_ recovery increases from 67.6 to 77.2% by increasing pH from 8 to 10, and then decreases to 76.1% at pH 11. Comparing the A/S and Al_2_O_3_ recovery treated by NaOl/TDM (pH = 9) and NaOl (pH = 10), the A/S in concentrate is 11.9 and 9.7, respectively, and the Al_2_O_3_ recovery for NaOl/TDM is 7.5% higher than that for NaOl. The flotation tests of mixed binary minerals further proves that mixed NaOl/TDM collector can better separate diaspore and kaolinite.

### Surface Tension Measurements

**Figure 7:**
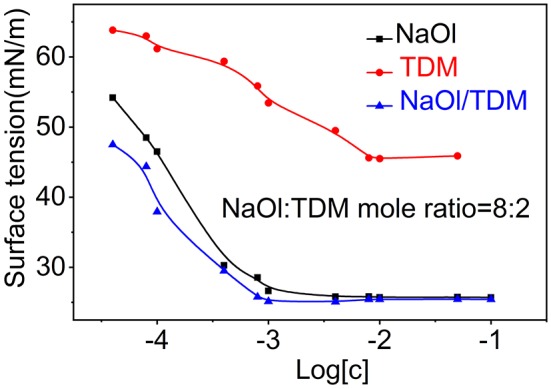
Surface tension for the individual collectors NaOl and TDM and mixed collector NaOl/TDM (NaOl: TDM mole ratio = 8:2).

The formation of micelles in solution and the reduction of surface tension are two important properties of surfactants. The critical micelle concentration (CMC) is one of the parameters to measure collector performance in flotation. Generally, the smaller the CMC is, the larger the proportion of non-polar groups in surfactants, which means that the agents have stronger hydrophobicity and stronger collectivity. The relationship between surface tension and concentration of individual collectors NaOl, TDM, and mixed collector NaOl/TDM is shown in Figure 7. The order of the CMCs and the lowest surface tension of the three surfactants in Table 1 is NaOl/TDM < NaOl < < TDM. The CMC and the lowest surface tension of TDM is the largest, so its hydrophobicity is the weakest. This also explains why flotation recovery of TDM is poor when it is used as single collector. The CMC of mixed NaOl/TDM collector is slightly smaller than that of NaOl. Therefore, NaOl/TDM is more hydrophobic than that of NaOl, which also explains why the flotation effect of mixed collector NaOl/TDM is better than that of NaOl. The free energy of micellization can be calculated from the CMC using Eq where R is the gas constant and T is the absolute temperature, with T set to 298.15 K when unknown (Butt et al., 2003).

**Table 1:** CMCs and related properties of NaOl, TDM, and NaOl/TDM.

**Surfactants**	**CMC(mmol/l)**	**The lowest surface tension(mN/m)**	**Micellization free energy (kJ/mol)**
NaOl	1	25.71	−22.82
TDM	8	45.51	−17.67
NaOl/TDM	0.8	25.09	−23.37

$$\begin{array}{l} \Delta G_{m}=\text{RTlnCMC } \end{array}$$

The free energy of micellization can reflect the strength of the hydrophobic association of surfactants. Generally, the lower the free energy of micellization of surfactants is, the stronger the hydrophobic association is Fan et al. (2015). The order of micellization free energy is TDM > NaOl > NaOl/TDM. The micellization free energy of mixed collector NaOl/TDM is the lowest, so its hydrophobic association and collecting ability is the strongest, which is consistent with the result of flotation.

### Zeta Potential Determinations

**Figure 8:**
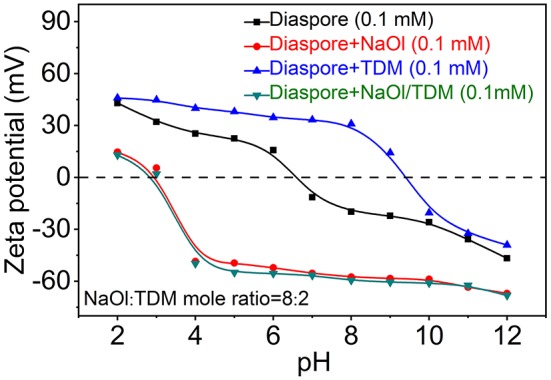
Zeta potentials of diaspore in the presence of various flotation reagents.

**Figure 9:**
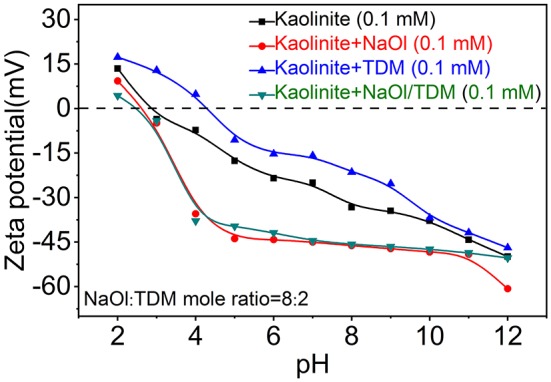
Zeta potentials of diaspore in the presence of various flotation reagents.

Zeta potentials of diaspore and kaolinite mixed with NaOl, TDM and NaOl/TDM collectors are shown in Figures 8, 9. Figures 8, 9 show that the IEP (isoelectric point) of diaspore and kaolinite obtained are ~6.6 and 3.2, respectively, which matches well with the previous reports (Guan et al., 2009; Liu et al., 2011; Huang et al., 2014; Lyu et al., 2019).

Compared with the Zeta potentials of diaspore and kaolinite in water, the Zeta potentials of diaspore and kaolinite mixed with single collector NaOl are shifted negatively, which indicates that NaOl is adsorbed on the surface of diaspore and kaolinite. Because NaOl has stronger adsorption and affinity to diaspore than kaolinite, the negative shift of Zeta potentials of diaspore is larger than that of kaolinite. The Zeta potentials of diaspore and kaolinite mixed with single collector TDM are moved in the positive direction, which indicates that TDM is adsorbed on the surface of minerals. However, the Zeta potential shift of diaspore and kaolinite in the presence of TDM is smaller than that of NaOl, which indicates that the adsorption of TDM on mineral surface is less than that of NaOl. This is consistent with the conclusion of flotation. The Zeta potentials of diaspore in NaOl/TDM mixed collector are slightly negatively shifted from those in NaOl solution, indicating that the addition of TDM can enhance the change of potentials. Unlike diaspore, the Zeta potentials of kaolinite in NaOl/TDM mixed collector are shifted slightly positively than those in NaOl solution, which indicates that the addition of TDM does not enhance the negative movement of Zeta potentials of kaolinite.

From Figure 8, we found that Zeta potential change of mineral surface is very small in the presence of NaOl and NaOl/TDM, which indicates that the change of Zeta potentials on mineral surface by mixed collector is limited, compared with NaOl. This may be due to the fact that TDM only accounts for 20% of the molar fraction of mixed collector, resulting in a limited increase in the amount of adsorption on the mineral surface. However, the addition of TDM can properly promote the adsorption of collector on diaspore surface and weaken the adsorption on kaolinite surface, so the selectivity of NaOl/TDM mixed collector is better than that of single NaOl collector.

### FTIR Spectra Analysis

In the infrared spectrum of NaOl, 2,957, 2,921, and 2,852 cm^−1^ are the stretching vibration absorption peaks of -CH_2_ in the hydrocarbon chain. The peak at 1,560 cm^−1^ belongs to the -COOC- asymmetric vibration. The peaks at 1,463 and 1,379 cm^−1^ are attributed to the symmetric stretching vibration of -COOC- (Wen et al., 2008; Wang et al., 2014). In the infrared spectrum of TDM, 2,926 and 2,852 cm^−1^ are the stretching vibration band of -CH_3_, 2,375 and 2,320 cm^−1^ are the -SH stretching vibration absorption peaks in sulfhydryl group. And spectra has bending vibration band of -CH_3_ and = CH_2_ in 1,560 and 1,463 cm^−1^ (Jiao et al., 2016). The FTIR spectra of NaOl and TDM are shown in Supplementary Material.

**Figure 10:**
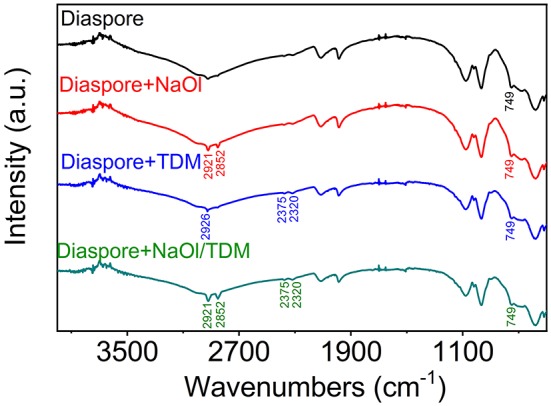
FTIR spectra of diaspore in different collectors (NaOl: TDM mole ratio = 8:2).

**Figure 11:**
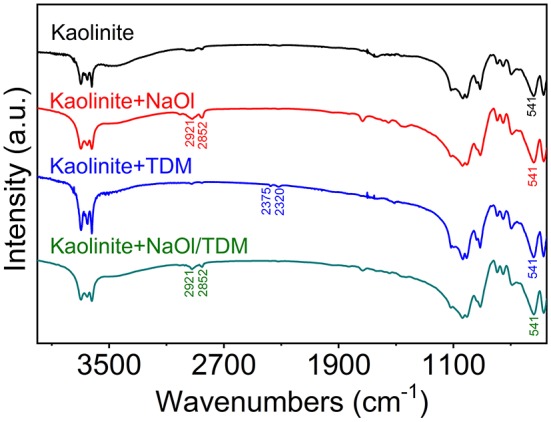
FTIR spectra of kaolinite in different collectors (NaOl: TDM mole ratio = 8:2).

Figures 10, 11 show the FTIR spectra of diaspore and kaolinite, which were in the presence of different collectors. It can be seen in Figure 10 that after the NaOl treatment, obvious -CH_2_ stretching vibration absorption peaks appear at 2,921 and 2,852 cm^−1^, which indicates that NaOl is adsorbed on the surface of diaspore. However, the Al-O stretching vibration peak at 749 cm^−1^ does not show obvious band shift, which indicates that NaOl is not adsorbed on the mineral surface by the chemical action of the Al atom site, but by physical adsorption. This is similar to the results of other scholars (Xia et al., 2009; Xu et al., 2014). Under the action of TDM, -SH stretching vibration absorption peaks appear at 2,375 and 2,320 cm^−1^. And stretching vibration band of -CH_3_ in 2,926 cm^−1^ appears. However, no band shift is observed, so it is presumed that TDM is physically adsorbed on the surface of diaspore. After the NaOl/TDM treatment, the appearance of -SH stretching vibration peaks (2,375 and 2,320 cm^−1^) indicates that TDM is adsorbed on the surface of diaspore. The appearance of -CH_3_ stretching vibration peak (2,921 and 2,852 cm^−1^) indicates that NaOl is also adsorbed on the mineral surface. Compared with the infrared spectra of diaspore treated by NaOl, the peak intensities treated by NaOl/TDM are strengthened, which indicates that diaspore treated by NaOl/TDM mixed collector has stronger adsorption. This is consistent with the conclusion of Zeta potential test.

As shown in Figure 11, the weak absorption vibration peaks of -CH_3_ appear at 2,921 and 2,852 cm^−1^ after the interaction between NaOl and kaolinite. No obvious band shift is found, which indicates that NaOl is also adsorbed on the mineral surface by physical action (Elbokl and Detellier, 2006; Xia et al., 2009). However, from the intensity of absorption peaks, its adsorptive effect is not as strong as that of NaOl on diaspore surface. A weak characteristic absorption peak of TDM appears in the infrared spectra of kaolinite treated by TDM, but the band shift is not observed, indicating that TDM is physically adsorbed on the mineral surface. After the treatment of NaOl/TDM, the characteristic peaks of NaOl show that it is adsorbed on the mineral surface, but no adsorption of TDM is found. It may be that the adsorption of TDM is weak, so the adsorption effect is not obvious at low concentration. Compared with the infrared spectra of kaolinite treated by NaOl, the peak intensities treated by NaOl/TDM are weakened, which indicates that diaspore treated by NaOl/TDM mixed collector has weaker adsorption. This is consistent with the flotation test results, which further proves that the mixed NaOl/TDM can effectively separate diaspore and kaolinite.

## Conclusion

In this work, tert dodecyl mercaptan (TDM) is first proposed as a mixed collector with NaOl for diaspore flotation. It is proved that the selective separation of diaspore and kaolinite can be effectively achieved by using mixed collector of NaOl and TDM (NaOl: TDM mole ratio = 8:2, dosage = 0.1 mM) at pH = 9. Compared with single collector NaOl or TDM, mixed collector NaOl/TDM has stronger hydrophobic association and surface activity. Mixed collector NaOl/TDM can co-adsorb on diaspore through physical adsorption, and the adsorption strength of diaspore surface is greater than that of kaolinite with NaOl/TDM mixed collector. The addition of TDM can enhance the adsorption strength of NaOl on diaspore. And TDM also adsorbs on the surface of diaspore, thus forming a synergistic adsorption between TDM and NaOl. For kaolinite, TDM has almost no adsorption on its surface when mixed collector NaOl/TDM treats together. These experimental results show that the addition of TDM can promote the flotation separation of diaspore from kaolinite by NaOl. Mixed collector NaOl/TDM can achieve better separation of diaspore and kaolinite.

## Data Availability Statement

All datasets generated for this study are included in the article/Supplementary Material.

## Author Contributions

XMan, LO, and CW conceived the research, designed the tests, and analyzed the data, while XMan, SJ, and XMa wrote and revised the manuscript.

### Conflict of Interest

The authors declare that the research was conducted in the absence of any commercial or financial relationships that could be construed as a potential conflict of interest. The handling editor declared a shared affiliation, though no other collaboration, with the authors LO, XMan, CW, SJ, and XMa at time of review.
