# Supplementary material for: Flotation Separation of Diaspore and Kaolinite by Using a Mixed Collector of Sodium Oleate-Tert Dodecyl Mercaptan
Source: Front Chem. 2019 Dec 12;7:813. doi: 10.3389/fchem.2019.00813 (PMC6922048; doi:10.3389/fchem.2019.00813)
Supplement: Supplementary file 1 [file Data_Sheet_1.ZIP › Revised supplementary material/Supplementary-Material.docx]

Supplementary Material

# Supplementary Figures and Tables

## Supplementary Figures


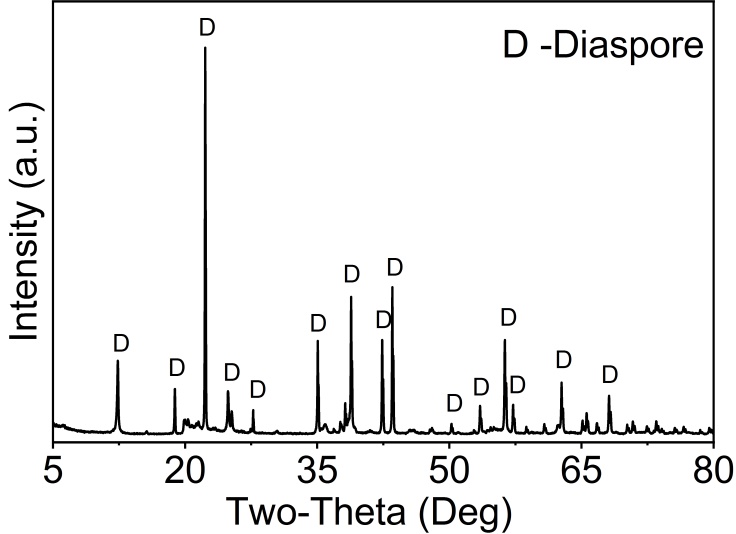


**Supplementary Figure 1.** **XRD pattern of pure diaspore mineral samples.**

**
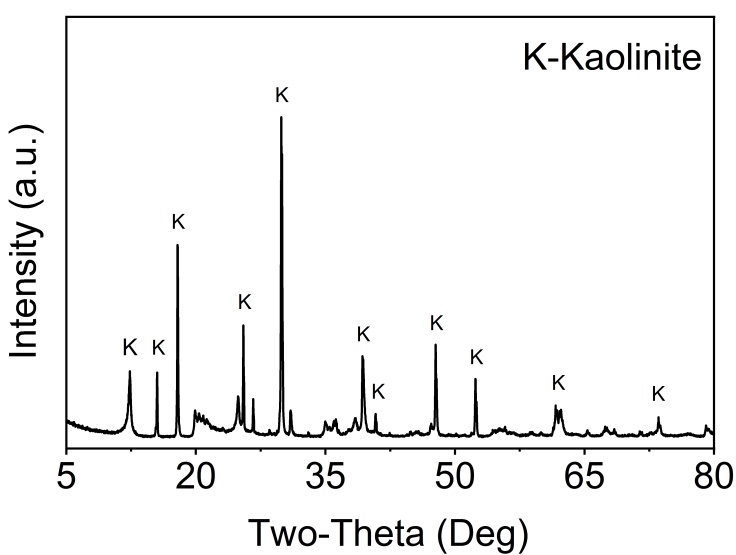
**

**Supplementary Figure 2. XRD pattern of pure kaolinite mineral samples.**

**
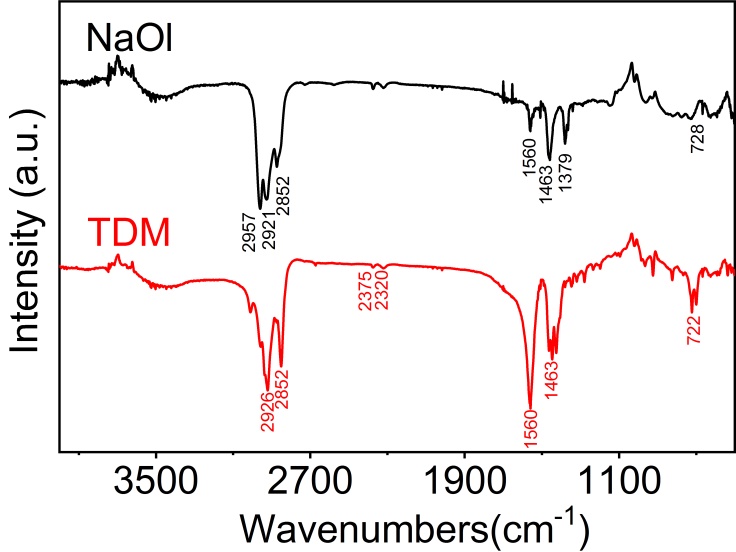
**

**Supplementary Figure 3. FTIR spectra of NaOl and TDM.**

**
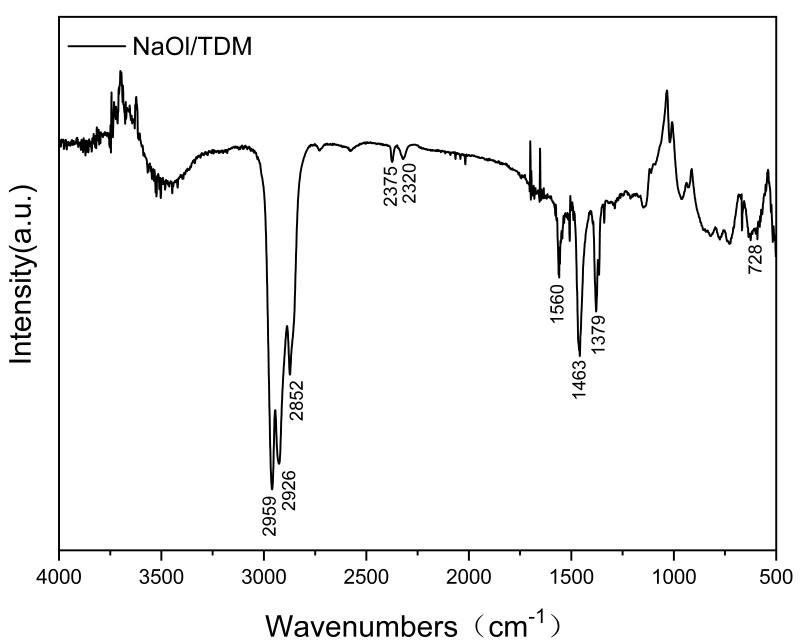
**

**Supplementary Figure 4. FTIR spectra of NaOl /TDM.**
